# Discovery and full genome characterization of two highly divergent simian immunodeficiency viruses infecting black-and-white colobus monkeys (*Colobus guereza*) in Kibale National Park, Uganda

**DOI:** 10.1186/1742-4690-10-107

**Published:** 2013-10-21

**Authors:** Michael Lauck, William M Switzer, Samuel D Sibley, David Hyeroba, Alex Tumukunde, Geoffrey Weny, Bill Taylor, Anupama Shankar, Nelson Ting, Colin A Chapman, Thomas C Friedrich, Tony L Goldberg, David H O'Connor

**Affiliations:** 1 Wisconsin National Primate Research Center, Madison, WI, USA; 2 Laboratory Branch, Division of HIV/AIDS Prevention, National Center for HIV, Hepatitis, STD, and TB Prevention, Centers for Disease Control and Prevention, Atlanta, GA, USA; 3 Department of Pathobiological Sciences, University of Wisconsin-Madison, Madison, WI, USA; 4 Makerere University, Kampala, Uganda; 5 Center for High-throughput Computing, University of Wisconsin-Madison, Madison, WI, USA; 6 Department of Anthropology and Institute of Ecology and Evolution, University of Oregon, Eugene, OR, USA; 7 Department of Anthropology and School of Environment, McGill University, Montreal, QC, Canada; 8 Wildlife Conservation Society, Bronx, NY, USA; 9 Department of Pathology and Laboratory Medicine, University of Wisconsin-Madison, 555 Science Drive, Madison 53711, WI, USA

**Keywords:** Simian immunodeficiency virus, SIV, Retrovirus, Lentivirus, Old World primate, *Colobus guereza*, Uganda, Next-generation sequencing, Virus discovery

## Abstract

**Background:**

African non-human primates (NHPs) are natural hosts for simian immunodeficiency viruses (SIV), the zoonotic transmission of which led to the emergence of HIV-1 and HIV-2. However, our understanding of SIV diversity and evolution is limited by incomplete taxonomic and geographic sampling of NHPs, particularly in East Africa. In this study, we screened blood specimens from nine black-and-white colobus monkeys (*Colobus guereza occidentalis*) from Kibale National Park, Uganda, for novel SIVs using a combination of serology and “unbiased” deep-sequencing, a method that does not rely on genetic similarity to previously characterized viruses.

**Results:**

We identified two novel and divergent SIVs, tentatively named SIVkcol-1 and SIVkcol-2, and assembled genomes covering the entire coding region for each virus. SIVkcol-1 and SIVkcol-2 were detected in three and four animals, respectively, but with no animals co-infected. Phylogenetic analyses showed that SIVkcol-1 and SIVkcol-2 form a lineage with SIVcol, previously discovered in black-and-white colobus from Cameroon. Although SIVkcol-1 and SIVkcol-2 were isolated from the same host population in Uganda, SIVkcol-1 is more closely related to SIVcol than to SIVkcol-2. Analysis of functional motifs in the extracellular envelope glycoprotein (gp120) revealed that SIVkcol-2 is unique among primate lentiviruses in containing only 16 conserved cysteine residues instead of the usual 18 or more.

**Conclusions:**

Our results demonstrate that the genetic diversity of SIVs infecting black-and-white colobus across equatorial Africa is greater than previously appreciated and that divergent SIVs can co-circulate in the same colobine population. We also show that the use of “unbiased” deep sequencing for the detection of SIV has great advantages over traditional serological approaches, especially for studies of unknown or poorly characterized viruses. Finally, the detection of the first SIV containing only 16 conserved cysteines in the extracellular envelope protein gp120 further expands the range of functional motifs observed among SIVs and highlights the complex evolutionary history of simian retroviruses.

## Background

Simian immunodeficiency viruses (SIV) are primate lentiviruses that naturally infect at least 45 different African non-human primate (NHP) species [1,2]. It is now well established that zoonotic transmission of SIVs from chimpanzees (*Pan troglodytes troglodytes*) and gorillas (*Gorilla gorilla gorilla*) as well as from sooty mangabeys (*Cercocebus atys*) led to the emergence of human immunodeficiency virus type 1 (HIV-1) and type 2 (HIV-2), respectively [3-5]. Despite high divergence among SIVs, each primate species is typically infected with one or more species-specific viruses. However, there are also numerous examples of cross-species transmission and recombination [6-10]. Interestingly, different animals from a single primate species can be infected by more than one SIV. For example, mandrills (*Mandrillus sphinx*) in Gabon and southern Cameroon are infected by two different SIVs, SIVmnd-1 or SIVmnd-2, possibly due to their geographic separation by the Ogoué River [8,11]. Even in the absence of physical barriers, two distinct SIVs can co-circulate in a single species living in a small geographical area, as observed for mustached monkeys (*Cercopithecus cephus*) in Cameroon that are infected by SIVmus-1 or SIVmus-2 [12]. Recently, it has been reported that mustached monkeys in Gabon are also infected with a highly divergent SIVmus, demonstrating that the same monkey subspecies can harbor at least three distinct SIVs [13].

Phylogenetic analysis shows that all SIVs cluster in a single clade within the mammalian lentiviruses, indicating descent from a single common ancestor [14]. Only African Old World monkeys and apes from sub-Saharan Africa, but not Asian Old World monkeys or New World monkeys, are naturally infected with SIV, suggesting that SIV originated in sub-Saharan Africa after the landmass separation and migration that gave rise to New World and Asian primate lineages [14,15]. Old World monkeys (*Cercopithecidae*) are separated into two distinct subfamilies, *Colobinae* and *Cercopithecinae*, which diverged approximately 18 million years ago (MYA) [16]. The *Colobinae* are further divided into African (*Colobini*) and Asian (*Presbytini*) groups and African colobines consist of two genera: *Colobus* and *Procolobus*[17]. SIVcol, isolated from a black-and-white colobus (*Colobus guereza*) in Cameroon, represents the only SIV infecting the *Colobus* genus for which full-length sequences are available [18], with partial sequences being available from black colobus (*Colobus satanas satanas*) from Bioko (49). The *Procolobus* genus includes red colobus (subgenus *Piliocolobus*) and olive colobus (subgenus *Procolobus*) and full-length SIV sequences are available from both groups from Tai forest in the Ivory Coast (*Procolobus badius badius* and *Procolobus verus*, respectively) [19]. In addition, SIV sequences are available from Tephrosceles red colobus (*Procolobus rufomitratus tephrosceles*) in Kibale National Park, Uganda [20], Temminck’s red colobus (*Procolobus badius temminckii*) from The Gambia [21] and Tshuapa red colobus (*Procolobus tholloni*) from the Democratic Republic of Congo [22], demonstrating the large geographic distribution and diversity of SIV in colobines. Interestingly, SIVcol is highly divergent from other known SIVs, possibly reflecting ancient divergence of host lineages.

**Figure 1:**
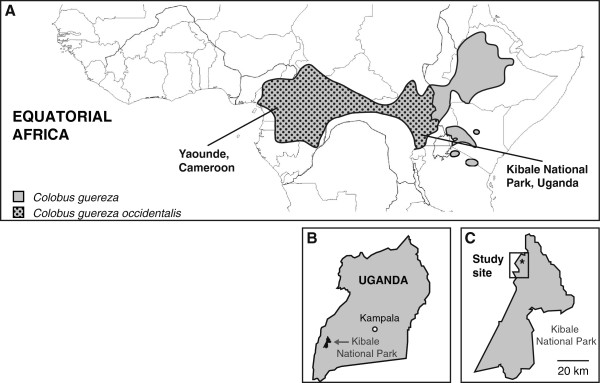
**Distribution of black-and-white colobus (*Colobus guereza*) across equatorial Africa. A**: *C. guereza* distribution is shown in grey and distribution of the *C. guereza occidentalis* subspecies is overlaid in black dots. The newly identified SIVkcol-1 and SIVkcol-2 were isolated from Kibale National Park, Uganda whereas the previously identified SIVcol was isolated from Yaounde, Cameroon. Map and distribution adapted from [44]. **B**: Location of Kibale National Park within Uganda. **C**: Location of the study site within Kibale National Park. Site of sample collection is indicated with a black star and encompasses an area of approximately 15 km^2^.

Colobine NHPs are distributed throughout equatorial Africa, however no full-length SIV sequences have been isolated from East African monkeys. Although the density and variety of East African NHP is high, particularly at Kibale National Park (KNP) in Uganda, this region has been undersampled [23]. To gain insight into the diversity of SIVs infecting *Colobus* monkeys from East Africa, we screened nine black-and-white colobus (*Colobus guereza occidentalis*) from KNP (Figure 1) for the presence of novel SIVs, the same park in which we previously recovered partial polymerase (*pol*) sequences from a divergent SIV infecting red colobus monkeys (*Procolobus rufomitratus tephrosceles*) [20]. Here, we report the discovery and full genome characterization of two novel SIVs identified in Kibale black-and-white colobus (*C. guereza*), tentatively named SIVkcol-1 and SIVkcol-2. These two viruses were discovered in three and four animals, respectively, using “unbiased” deep-sequencing, a method that does not rely on homology to previously characterized genomes and is thus more sensitive for detecting divergent sequences. Furthermore, we establish phylogenetic relationships to other known SIVs, and functionally characterize both viruses.

## Results

### Discovery of novel SIVs from KNP, Uganda

Blood plasma from nine black-and-white colobus monkeys (BWC) from Kibale National Park, Uganda, (Figure 1) was screened for the presence of SIVs using a combination of deep-sequencing and serology. The sequencing approach applied in this study is “unbiased” in that it uses random hexamers for priming and therefore does not rely on homology to known sequences. This approach is therefore less biased than specific PCR and may be more likely to discover novel and divergent viruses, as previously demonstrated by the discovery of other novel RNA viruses in monkeys from the same national park [24-26]. On average, around 456,000 reads per sample were generated. A query of reads against the GenBank database using the basic local alignment search tool blastn [27], locally implemented on the University of Wisconsin-Madison’s Condor High Throughput Computing cluster, revealed the presence of SIV reads in seven of nine animals, ranging from 0.2% (2,220 reads) to 7.9% (79,375 reads) of total reads (Table 1). For two of those animals (BWC01 and BWC07), enough reads were present to *de novo* assemble SIV genomes covering the entire coding region. A query against the NCBI GenBank database [27] revealed that the two viruses shared between 73% and 77% nucleotide identity with SIVcol, an SIV that was previously discovered in black-and-white colobus monkeys from Cameroon [18]. Furthermore, a pairwise comparison between the two new viruses revealed that the genomes were distinct from each other, sharing only 72% nucleotide identity (Table 2). This is comparable with the two distinct SIV variants infecting mustached monkeys in Cameroon, SIVmus-1 and SIVmus-2, which share 73% nucleotide identity. For consistency with established nomenclature, both viruses were tentatively named SIVkcol-1 and SIVkcol-2, reflecting their origin from KNP as well as their relation to SIVcol [GenBank sequence accession numbers KF214240 and KF214241]. To determine the frequency of SIV infection for each variant, we mapped reads to the previously assembled SIVkcol-1 and SIVkcol-2 genomes. Among the nine black-and-white colobus, three were infected with SIVkcol-1 and four with SIVkcol-2. Interestingly, no co-infections were observed in any BWC in our study and none of the variants was restricted to any one social group. The sequence of the *de novo*-assembled SIVkcol-1 and SIVkcol-2 genomes was confirmed by generating four overlapping amplicons covering the entire ORF followed by deep-sequencing on the Illumina MiSeq. Both SIVs contain genomic structures similar to those of complex retroviruses, including all three structural (*gag, pol* and *env*) as well as various accessory genes (*vif, vpr, tat, rev* and *nef*), thus resembling the genome organization of SIVcol. No additional accessory genes previously reported for other SIVs, like *vpx* or *vpu*, were present in either of the two novel BWC SIVs [1].

**Table 1:** **Prevalence of SIVs in black-and-white colobus (BWC) monkeys at Kibale National Park, Uganda**^**1, 2**^

**Animal**	**Sex**	**HIV-2 WB**		**HIV-1/2 InnoLIA**				**SIV deep-sequencing (# of SIV reads)**^**3**^
		**Gag**	**Pol**	**Env**	**Gag**	**Pol**	**Env**	
BWC01	M	+ (p26)	-	-	-	-	-	SIVkcol-1 + (79375)
BWC02	M	-	-	-	-	-	-	SIVkcol-2 + (11408)
BWC03	F	+ (p26)	-	-	-	-	1+ (gp36)	SIVkcol-2 + (6752)
BWC04	F	-	-	-	-	-	-	SIVkcol-2 + (3267)
BWC05	M	-	-	-	-	-	-	SIVkcol-1 + (5826)
BWC06	F	-	-	-	-	-	-	-
BWC07	F	-	-	-	-	-	-	SIVkcol-2 + (2220)
BWC08	M	-	-	-	-	-	-	SIVkcol-1 + (10741)
BWC09	F	-	-	-	-	-	-	-

^1^The prevalence of SIV infections in black-and-white colobus monkeys (BWC) was assessed by deep-sequencing of reverse transcribed viral RNA and with serological tests, including HIV-2-specific western blots (HIV-2 WB) and HIV InnoLIA assays.

^2^For HIV-2 WB and HIV-1/-2 InnoLIA, seroreactivity to specific viral proteins is shown in brackets. For the HIV-1/-2 InnoLIA assay, intensity of signals was quantified with 1+ being positive and 3+ being strongly positive.

^3^To quantify the number of SIV-specific reads, read number were normalized to 1,000,000 million reads per animal.

**Table 2:** Percent nucleotide identity across the coding region of different colobine SIVs

**Strain**	**SIVkcol-1**	**SIVkcol-2**	**SIVcol**	**SIVwrc**	**SIVolc**
**SIVkcol-1**	100				
**SIVkcol-2**	72.2	100			
**SIVcol**	76.3	71.8	100		
**SIVwrc**	49.5	48.7	48.8	100	
**SIVolc**	50.5	49.5	49.3	58.9	100

Antibody reactivity against SIV proteins was observed in two of nine BWC (Table 1). In the HIV-2 specific WB, BWC01 and BWC03 showed antibody responses whereas in the HIV-1/-2 InnoLIA assay, only the plasma of BWC03 was weakly seroreactive. While antibodies in the HIV-2 WB were specific for the p26 matrix protein, antibodies in the HIV-1/-2 InnoLIA assay reacted against the HIV-2 Env protein gp36 (Table 1). Both animals that showed seroreactivity were SIV-RNA positive, but we were also able to recover SIV-specific reads from five additional animals that did not show any antibody reactivity in these two assays. Taken together, the random hexamer-based detection of SIV-RNA in blood was more sensitive than the detection of cross-reactive antibodies to HIV-1 or HIV-2 proteins using the HIV-2 WB and HIV-1/-2 InnoLIA assays and suggests that prevalence estimates solely based on those methods could underestimate overall SIV prevalence.

### Functional motifs in Gag and Env proteins

**Figure 2:**
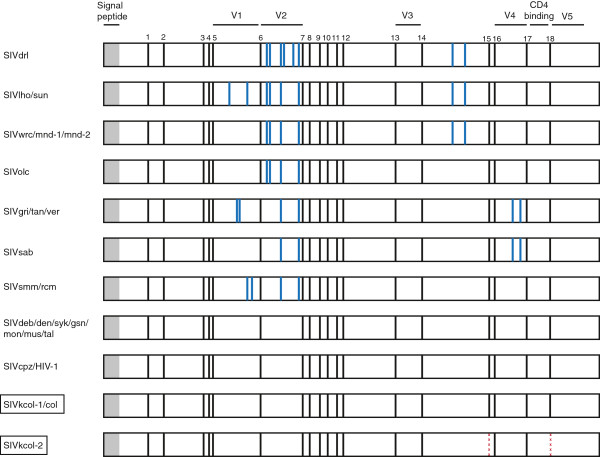
**Location of cysteine pairs in the extracellular envelope glycoprotein gp120 of primate lentiviruses.** Signal peptide, variable regions 1–4 (V1-V4) as well as the CD4-binding site are shown in relation to their position within gp120. Black bars indicate the position of the 18 conserved cysteine residues while blue bars indicate additional cysteines. The position of the missing cysteines for SIVkcol-2 is highlighted with a dashed red line.

All primate lentiviruses characterized to date contain at least 18 conserved cysteine residues in the extracellular subunit of gp120, however SIV strains can also contain up to four additional cysteine pairs, generally located in the variable domains of gp120 (Figure 2) [15]. SIVcol, isolated from a black-and-white colobus from Cameroon, contains 18 conserved cysteine residues with no additional cysteine pairs (Figure 2). While the same cysteine architecture was also conserved for SIVkcol-1, SIVkcol-2 only contains 16 conserved cysteine residues in the extracellular gp120. Specifically, conserved cysteine residues 15 and 18 in the C-terminal half of Env, known to form a disulfide bond in HIV-1, were missing [28]. The same unusual cysteine architecture was conserved among all four SIVkcol-2 positive animals (BWC02, BWC03, BWC04, BWC08).

PT/SAP and YPXL are two binding site motifs within the SIV Gag p6 protein that have been identified to be crucial for lentiviral budding [29-31]. The presence of one motif can compensate for the absence of the other, but both motifs can also be present at the same time [32,33]. Both SIVkcol-1 and SIVkcol-2 are missing the PT/SAP motif and only contain a singular YPXL motif, thus resembling SIVcol.

### Sequence similarity and phylogenetic analyses

**Figure 3:**
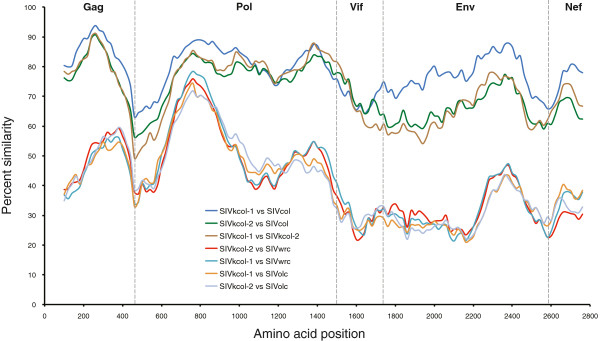
**Sliding window similarity plots of concatenated protein sequences of SIVkcol-1 and SIVkcol-2 against other *Colobine *SIVs.** Dashed vertical lines indicate start positions of viral proteins Gag, polymerase (Pol), Vif, envelope (Env), and Nef.

In order to compare genomes of the two newly discovered SIVs from KNP to other previously characterized SIVs infecting the *Colobini*, we performed a similarity analysis of concatenated Gag, Pol, Vif, Env and Nef protein sequences (Figure 3). Across the genome, SIVs infecting BWC monkeys (SIVkcol-1, SIVkcol-2 and SIVcol) are more similar to each other than to those infecting red and olive colobus (SIVwrc and SIVolc, respectively), possibly reflecting divergence between the host genera *Colobus* and *Procolobus* (Table 2). Interestingly, although SIVkcol-1 and SIVkcol-2 were both isolated from BWC monkeys from Kibale, SIVkcol-1 is consistently more similar to SIVcol from Cameroon than to SIVkcol-2, particularly in Env. Confirming results from previous studies, a 200 aa region in the N-terminal half of Pol (approximately positions 700 to 900 in the simplot alignment) shares the highest similarity between the *Colobus* and *Procolobus* SIVs [18,19]. Using the NCBI conserved domain and protein classification database, we identified this region as the reverse transcriptase (RT) domain (cd01645) [34].

Pairwise comparisons of nucleotide identity across the entire coding region further illustrate the divergence between SIVs isolated from *Colobus* and *Procolobus* as well as the closer relationship of SIVkcol-1 and SIVcol (Table 2). Although we were unable to assemble full SIV genomes for every infected animal, we obtained consensus sequences covering the entire Gag protein from all seven SIV-infected BWC colobus monkeys (three infected with SIVkcol-1, four infected with SIVkcol-2), allowing us to further assess inter-host genetic diversity between different variants of the same virus [GenBank sequence accession numbers KF214242-KF214246]. Overall, SIVkcol-1 was slightly more diverse than SIVkcol-2, sharing 88.7 ± 5.3% nucleotide identity among strains (Table 3), whereas isolates of SIVkol-2 were 93.9 ± 5.9% identical (Table 4). We also identified two SIVkcol-2 strains (from BWC03 and BWC07) exhibiting ≤2% divergence across Gag, potentially indicating epidemiologically linked infections [35]. The fact that both animals belonged to the same social group further supports the idea that close contact between those two animals resulted in direct transmission of the virus.

**Table 3:** Percent nucleotide identity for SIVkcol-1 infected black and white colobus (BWC) across gag

	**BWC01**	**BWC05**	**BWC08**
**BWC01**	100		
**BWC05**	85.7	100	
**BWC08**	85.7	94.8	100

**Table 4:** Percent nucleotide identity for SIVkcol-2 infected black and white colobus (BWC) across gag

	**BWC02**	**BWC03**	**BWC04**	**BWC07**
**BWC02**	100			
**BWC03**	97.4	100		
**BWC04**	87.5	87.5	100	
**BWC07**	97.5	**99.7**	87.5	100

Percent identities above 98 are highlighted in bold.

**Figure 4:**
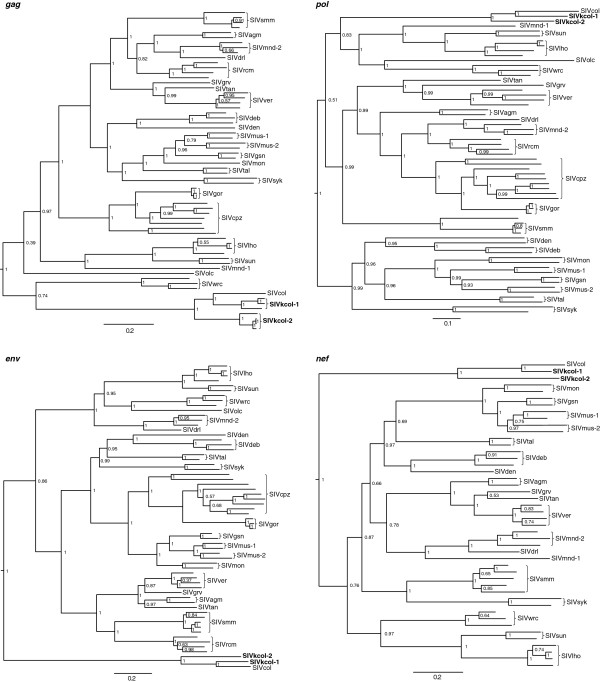
**Phylogenetic relationships of newly discovered SIVkcol-1 and SIVkcol-2 to other SIVs.** Separate Bayesian Markov Chain Monte Carlo phylogenies were constructed for *gag*, polymerase (*pol*), envelope (*env*) and *nef* proteins. Posterior clade probabilities are shown on branches. The scale bar below the phylogenetic trees represents substitutions per site. SIVkcol-1 and SIVkcol-2 are highlighted in bold.

To estimate phylogenetic relationships of the two novel SIVs to other known SIVs, we constructed separate evolutionary trees for *gag, pol, env* and *nef* genes. In all four phylogenies, SIVkcol-1 and SIVkcol-2 formed a highly supported distinct lineage with SIVcol, with SIVkcol-2 in a distinct branch ancestral to SIVcol and SIVkcol-1, similar to the genetic relationships described above in the similarity plot analysis (Figure 4). In the *env* and *nef* trees, the ancestor of the BWC SIVs diverged at the root of the SIV tree. In the *gag* tree, the colobus SIVs clustered weakly with procolobine SIVs from western red colobus, while the other procolobine SIV, SIVolc, clusters with all other SIVs, though with low posterior support. In the *pol* trees, the colobus SIVs share a common ancestor with the procolobine SIVs (SIVolc and SIVwrc) and the SIVsun/l’hoest and SIVmnd lineages.

**Figure 5:**
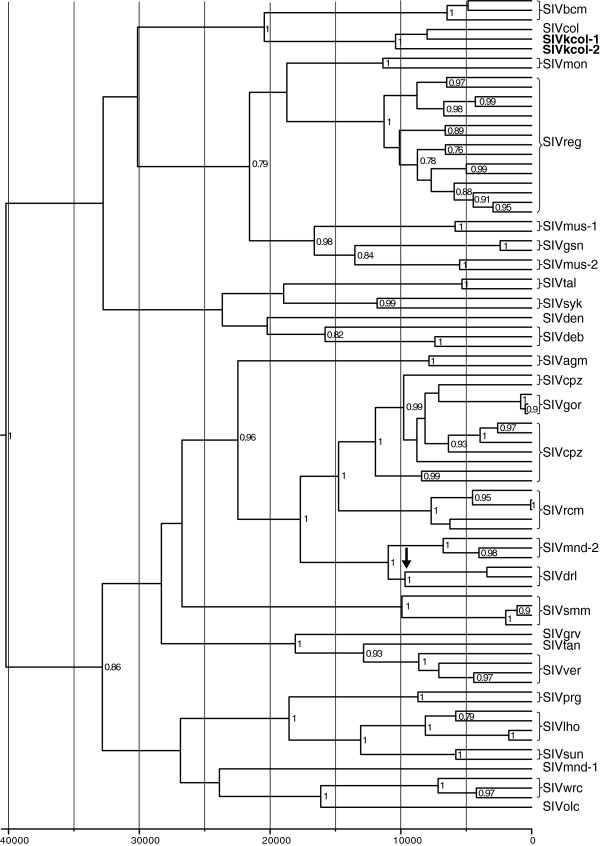
**Time to most recent common ancestor (TMRCA) for SIVkcol-1 and SIVkcol-2 and other representative SIVs.** Bayesian Markov Chain Monte Carlo phylogenies were generated in order to estimate TMRCA. The scale bar below the phylogenies represents years before present and the black arrow represents the Bioko calibration point used in this analysis. Posterior clade probabilities above 0.7 are shown on branches. SIVkcol-1 and SIVkcol-2 are highlighted in bold.

To estimate the age of the novel SIVs in relation to other SIVs, we determined TMRCA using Bayesian inference. The relaxed molecular clock used in this analysis was based on the adjusted SIV substitution rate that was previously determined for divergence of the Bioko monkey SIVs and used a 308-bp alignment of conserved *pol* sequences [36]. The root of the tree is estimated to be 40,323 years before present (ybp) (95% highest posterior density (HPD) = 24,406 - 61,988 ybp) and is thus comparable to that of inferred for the Bioko monkey SIV phylogenies (49,129 ybp; 95% HPD = 29,078 - 71,268 ybp) (Figure 5, Table 5). The split between SIVkcol-1/col and SIVkcol-2 occurred at least 10,657 ybp (95% HPD = 5,215-18,146 ybp). Despite the use of a strong geological calibration point for our molecular dating estimates, we acknowledge that considerable debate exists about the accuracy of SIV TMRCA estimates and suggest that dates should be regarded as minimum estimates [36].

**Table 5:** **Time to most recent common ancestor (TMRCA) for newly discovered SIVs and other related SIV lineages**^**1**^

**Clade**	**TMRCA**^**2**^	**95% HPD**
SIV root	40,330	23,804 – 63,190
SIVdrl(mainland)/SIVdrl(Bioko)	9,607	7,671 – 11,606
SIVbcm/SIVcol/SIVkcol	20,560	10,191 – 34,569
SIVcol, SIVkcol-1, SIVkcol-2	10,461	4,961 – 18,062
SIVolc/SIVwrc	7,904	3,560 – 13,684

^1^The separation of mainland drills from those SIVs on Bioko Island, Equatorial Guinea of about 10,000 years before present (ybp) was used as a calibration point for the molecular dating of SIVs with Bayesian inference in the program BEAST.

^2^TMRCAs are in ybp.

## Discussion

Although colobine primates are distributed throughout equatorial Africa, no full-length SIVs from this subfamily have been obtained from East Africa, potentially influencing our current understanding of the diversity and evolutionary history of SIV. We therefore screened nine BWC monkeys (*Colobus guereza occidentalis*) from KNP in Uganda, a park known for its exceptionally high density and diversity of NHPs [23], for the presence of SIVs using a combination of deep-sequencing and serological testing. Here, we report the discovery and characterization of two novel SIVs, tentatively named SIVkcol-1 and SIVkcol-2, in three and four animals, respectively. The new viruses are divergent from each other as well as from the previously discovered SIVcol from Cameroon and are both circulating within the same host population in KNP.

Traditionally, methods to recover complete or partial SIV genomes have relied on PCR using consensus primers. The design of those primers is based on regions conserved among different SIV lineages and amplified products are used to further characterize the virus and to confirm serological results. Here, we report for the first time a random hexamer based deep-sequencing approach to identify novel SIVs. This approach does not rely on homology to known sequences and is thus more sensitive for detecting divergent sequences, as previously demonstrated by the discovery of other novel RNA viruses in monkeys from the same national park [24-26]. Within the BWC population at KNP, no animals were found to be co-infected with SIVkcol-1 and SIVkcol-2. While we cannot exclude that this is due to the small sample size, infection with one virus could also protect against infection with the other, potentially through establishment and cross-reactivity of adaptive immune responses [37]. Additional sampling as well as *in vitro* experiments will be necessary to clarify the interactions between these viruses.

There was a strong discordance between infection data obtained by random hexamer-based deep-sequencing and serological testing, with 7/9 and 2/9 of animals being vRNA- and antibody-positive, respectively. One possible explanation could be the high divergence of SIVkcol-1 and SIVkcol-2 to HIV-1 and HIV-2, thus limiting cross-reactivity with HIV antigens used in the HIV-2 WB and the HIV-1/-2 InnoLIA assays. Similar serological results were observed for SIVcol-infected BWC from Cameroon supporting this hypothesis [18]. Recently, SIV lineage-specific ELISAs and flow-cytometry based assays have been successfully employed to detect specific SIV antibody responses [22,38]. Although these assays might have higher specificity, they must be regularly updated when new lineages are discovered and not every lineage-specific peptide can be successfully synthesized [22]. The serological assays used in this study have been successfully applied in the past to detect infection with divergent SIVs [38-41], however we also acknowledge that SIVcol lineage-specific assays might have resulted in a higher sensitivity compared to the serological assays employed in our study.

Another explanation for the low frequency of serological detection could be that the majority of BWC were acutely infected with SIV and thus had not mounted an antibody response at the time of sampling, although this might be unlikely given that samples were collected over a range of more than seven months. Overall, we believe that the use of blood as a sample source in combination with random hexamer-based deep-sequencing allows for a reliable assessment of SIV infection in wild NHP. Furthermore, studies relying only on serological data to either determine SIV prevalence or to discover new viruses may potentially be underestimating the occurrence and diversity of those viruses, particularly for NHPs infected with highly divergent SIVs.

BWC are the third NHP species found to be infected with two distinct SIV variants. Mandrills were the first species reportedly infected with two different SIVs. However, mandrill populations harboring SIVmnd-1 and SIVmnd-2 were geographically separated by the Ogoué River in Gabon, possibly explaining the presence of two divergent variants within this population [8,11]. A second species, mustached monkeys (*C. cephus*) from Cameroon, are also infected by at least two distinct viral variants. Interestingly, SIVmus-1 and SIVmus-2 were detected in samples collected within a radius of 5 km which is comparable to the area in which we collected samples from BWC in Kibale, thus providing a second example for SIVs co-circulating within a geographically confined NHP population.

The newly discovered SIVkcol-1 and SIVkcol-2 are most closely related to SIVcol and form a BWC-specific SIV lineage. For the 3′ genomic region (*env* and *nef*), the BWC SIVs originate near the root of the SIV tree, suggesting that SIV was introduced to the *Colobus* genus after the divergence from the *Procolobus*. In contrast, in the *gag* region the BWC SIVs cluster weakly with the procolobine SIV from western red colobus monkeys, but both lineages are divergent from the procolobine SIV in olive colobus monkeys. Likewise, in the *pol* gene the BWC SIVs share a common ancestry with *Procolobus*-infecting SIVs and the SIVsun/lst and SIVmnd-1 lineages, although this observation should be viewed with caution due to the high degree of divergence characterized by the long branch length. Furthermore, the high similarity observed by similarity plot analysis in the N-terminal half of Pol between *Colobus* and *Procolobus* genera might not necessarily be reflective of a common ancestry but rather represent high conservation of the essential RT domain, thereby obscuring ancestral relationships. Sequences from additional colobus and procolobus monkeys may be needed to further clarify the phylogenetic relationships of the 3′ genomes of colobine SIVs and their ancestral origins.

Surprisingly, although both SIVkcol-1 and SIVkcol-2 were isolated from the same group of BWC, SIVkcol-1 is more closely related to SIVcol from Cameroon. Since all three strains were isolated from the same subspecies (Figure 1), recent gene flow between the different strains as well as ancestral polymorphisms could explain their unusual relationship. These explanations are particularly feasible given the large population size and relatively continuous range of this subspecies across Central Africa. Based on our estimates, SIVcol and SIVkcol-1 diverged from SIVkcol-2 around 10,600 ybp, potentially explaining the high amount of divergence observed between these viruses. More sampling, covering populations across the range of this subspecies (*C. guereza occidentalis*) as well as other *C. guereza* subspecies across equatorial Africa, should resolve uncertainties about the evolutionary history of SIVs infecting this species.

Two different binding site motifs within the SIV Gag p6 protein have been identified to be crucial for lentiviral budding: PT/SAP and YPXL [29-31]. While the presence of one motif can compensate for the absence of the other, both motifs can also be present at the same time, although the significance of maintaining two binding site motifs is unknown [32,33]. The majority of SIVs contain either both motifs or a singular PT/SAP motif in the Gag p6 protein [21]. Only three viruses, SIVdeb from De Brazza’s monkeys (*Cercopithecus neglectus*), SIVden from Dent’s monkeys (*Cercopithecus denti*) and SIVcol from black-and-white colobus, have a singular YPXL motif. Both SIVkcol-1 and SIVkcol-2 are missing the PT/SAP motif and only have the YPXL motif, thereby confirming the singular presence of the YPXL motif among *Colobus*-infecting SIVs while also expanding the number of viruses exclusively using this budding motif.

All known primate lentiviruses characterized to date contain at least 18 conserved cysteine residues in the extracellular subunit of the envelope protein and this is also referred to as the “18 Cys state” [15]. Covalent disulfide bridges formed by cysteine pairs determine the tertiary structure of gp120 and are therefore essential for envelope function, including binding to the host cell receptor CD4 [28]. We believe that SIVkcol-2 is the first primate lentivirus that only contains 16 cysteines (Figure 2). Bibollet-Ruche *et al.* have speculated that the conservation of 18 cysteine residues across all SIVs represents an ancestral state of primate lentiviruses and that different SIV lineages have eventually added cysteine pairs over time [15]. The original “18 Cys state” has been independently conserved in *Cercopithecus* SIVs, SIVcpz (which is a recombinant containing a *Cercopithecus* envelope), as well as in SIVcol and SIVkcol-1. Currently, we are uncertain what led to SIVkcol-2 having only 16 cysteine residues and whether this is a result of “losing” a cysteine pair or whether the “16 Cys state” existed before the “18 Cys state”, thus represents an ancient state. Further sampling of BWC as well as other NHP will be required to confirm the presence of the “16 Cys state” in other NHP species. Additional studies will also be required to determine if the missing disulfide bond affects the antigenic structure of gp120.

## Conclusions

Our results demonstrate that SIV diversity in black-and-white colobus is greater than previously appreciated, and that divergent SIVs can co-circulate in the same colobine population. The success of our of “unbiased” molecular detection methods and our finding of two novel viruses in East African NHP indicate that using similar methods in similarly under-sampled settings is likely to be a fruitful avenue for future research. Also, our results show that deep sequencing has some advantages over traditional serological approaches, especially for the detection of unknown or poorly characterized viruses. Additional sampling of BWC across Africa is needed to confirm both the ubiquity of the unusual cysteine architecture observed in the envelope of SIVkcol-2, and the full extent of phylogenetic diversity among SIVs infecting the colobine primates.

## Methods

### Sample collection

All animal procedures followed the guidelines of the Weatherall Report on the use of NHPs in research and received approval from the Uganda Wildlife Authority, the Uganda National Council for Science and Technology, and the University of Wisconsin Animal Care and Use Committee, prior to initiation of the research, and materials were shipped in accordance with international regulations (CITES permit 002290). The study was conducted in KNP, western Uganda, a semi-evergreen, montane forest (795 km^2^) at the foothills of the Rwenzori Mountains, notable for its diversity and density of NHPs. As part of a larger study of wild primate health and infection, from January 27th to July 08th 2010, nine black and white colobus monkeys (all adult or subadult) were anesthetized with a combination of ketamine (5.11 ± 1.79 mg/kg) and xylazine (1.05 ± 0.12 mg/kg) or medetomidine (0.10 ± 0.08 mg/kg) administered intramuscularly using a variable-pressure air rifle (Pneudart, Inc, Williamsport, PA, USA) [42]. Blood was drawn from the femoral vein into an evacuated plasma preparation tube (Becton, Dickinson and Company, Inc, Franklin Lakes, NJ, USA) and kept cool until processing. Animals were then given the reversal agent atipamezole (0.32 ± 0.19 mg/kg) and released after recovery back to their social group without incident. Seven of the nine black-and-white colobus belonged to five different social groups with overlapping home ranges [43]. The social groups of the remaining two monkeys were unknown. All samples were collected within an area of approximately 15 km^2^. Blood was separated into components using centrifugation in a field laboratory and frozen immediately in liquid nitrogen for storage and transport.

### RNA extraction and deep-sequencing

From each animal, one ml of plasma was centrifuged at 5,000 × g (4°C, 5 min) with subsequent filtration of the supernatant through a 0.45-μm filter (Millipore, Billerica, MA, USA) to remove residual host cells. Viral RNA was then isolated using the Qiagen QIAamp MinElute virus spin kit (Qiagen, Hilden, Germany) according to the manufacturer’s instructions, but omitting carrier RNA. The eluted RNA was treated with DNase I (DNA-free, Ambion, Austin, TX, USA) and double stranded DNA was generated using the Superscript double-stranded cDNA Synthesis kit (Invitrogen, Carlsbad, CA, USA), primed with random hexamers. The DNA was purified using the Agencourt Ampure XP system (Beckman Coulter, Brea, CA, USA) and approximately one ng of DNA was subjected to simultaneous fragmentation and adaptor ligation (“tagmentation”) with the Nextera DNA Sample Prep Kit (Illumina, San Diego, CA, USA). One sample (BWC01) was also subjected to tagmentation with the Roche/454 Titanium-compatible Nextera kit (Epicentre Biotechnologies, Madison, WI, USA). DNA was subsequently cleaned using the Agencourt Ampure XP system, PCR amplified (15 cycles) to add Illumina- and Roche/454-compatible adaptors onto each fragment, and cleaned again with the Agencourt Ampure XP system. DNA fragments were then sequenced using the Illumina MiSeq (Illumina, San Diego, CA, USA) or the Roche/454 GS Junior instruments (Roche 454 Life Sciences, Branford, CT, USA).

Sequence data were analyzed using CLC Genomics Workbench 5.5 (CLC bio, Aarhus, Denmark). Low quality (<q30) and short reads (<100 bp) were removed and the remaining reads were subjected to *de novo* assembly using the following parameters: automatic word and bubble size; mismatch cost = 2; insertion cost = 3; deletion cost = 3; length fraction = 0.5; similarity fraction = 0.8. Assembled contiguous sequences (contigs) and singleton reads were queried against GenBank databases nt and nr using the basic local alignment search tools blastn and blastx, respectively.

In order to deep-sequence SIV genomes in infected individuals, we designed four overlapping PCR amplicons of approximately 2.5 kb each covering the entire SIV coding genome. Primers for each amplicon were based on the sequences obtained through de novo assembly of singleton reads. Viral RNA was prepared from 1 ml of plasma as described above, except that carrier RNA was added during the extraction. Viral RNA was then reverse transcribed and amplified using the SuperScript III High Fidelity One-Step RT-PCR kit (Invitrogen, Life Technologies, Carlsbad, CA). The reverse transcription-PCR conditions were as follows: 50°C for 30 min; 94°C for 2 min; 40 cycles of 94°C for 15 sec, 55°C for 30 sec, and 68°C for 3 min; and 68°C for 5 min. Following PCR, amplicons were purified from excised gel slices (1% agarose) using a Qiagen MinElute Gel Extraction kit (QIAGEN, Valencia, CA). Each amplicon was quantified using Quant-IT HS reagents (Invitrogen, Life Technologies, Carlsbad, CA), and all amplicons from a single viral genome were pooled together at equimolar ratios. Each pool was then quantitated and approximately 50 ng of each was used in a tagmentation reaction with Nextera DNA Sample Prep Kit (Illumina, San Diego, CA, USA). Final libraries representing each genome were characterized for average size using a DNA high sensitivity chip on a 2100 bioanalyzer (Agilent Technologies, Loveland, CO) and quantitated with Quant-IT HS reagents. Libraries were sequenced on the Illumina MiSeq as described above. Primer sequences employed are available upon request.

### Serological assays

Plasma samples were screened for HIV/SIV antibodies by using the Innogenetics INNO-LIA HIV-1/-2 Score assay (Innogenetics NV, Gent, Belgium) capable of detecting SIVcpz; HIV-1 groups M, N, and O; and other divergent SIVs [38-41]. The following recombinant proteins and peptides are used as antibody targets in the INNO-LIA assay: sgp120 (HIV-1); gp41 (HIV-1); p31, p24, p17 (HIV-1 proteins capable of cross-reacting with HIV-2 antibodies); sgp105 (HIV-2); gp36 (HIV-2). Samples were further tested for the presence of antibodies by using an HIV-2-based Western blot (WB) test (MP Biomedicals,Singapore), targeting the following proteins and peptides: gp125, gp80, p68, p56, p53, gp36 and p26. Both serologic assays have been previously shown to have good sensitivity in identifying divergent SIVs [38,39,41].

### Viral sequence and phylogenetic analyses

Nucleotide sequences of *gag*, *pol*, envelope (*env*) and *nef* were codon aligned individually for all known SIVs with complete genomes using ClustalW in the alignment editor program in MEGA v5.10 and edited manually. The best fitting distance model of nucleotide substitution for each alignment was inferred using the maximum likelihood (ML) method with goodness of fit measured by the Bayesian information criterion in MEGA v5.10. The best fitting nucleotide substitution model for the phylogenetic alignments was inferred to be the GTR model with discrete gamma and invariant among-site rate variation. Phylogenetic relationships were inferred using Bayesian analysis with the BEAST v1.6.2 program [44]. Statistical support for the inferred Bayesian trees was assessed by posterior probabilities. For the Bayesian analyses, an uncorrelated lognormal, relaxed molecular clock model was used and each run consisted of two independent 50 × 10^6^ Markov chain Monte Carlo (MCMC) generations with sampling every 5,000 generations and a Yule coalescent tree prior. Convergence of the MCMC was assessed by calculating the effective sampling size (ESS) of the runs using the program Tracer v1.5 (http://beast.bio.ed.ac.uk/Tracer). All parameter estimates showed significant ESSs > 1,200. The tree with the maximum product of the posterior clade probabilities was chosen from the posterior distribution of 10,001 sampled trees after burning in the first 1,000 sampled trees with the program TreeAnnotator version 1.6.2. The amino-acid similarity of the novel SIVs with related SIV lineages was determined across Gag, Pol, Env and Nef using SimPlot v3.5.1 (ref) following TranslatorX alignment (MAAFT) without Gblocks cleaning.

Time to the most recent common ancestor (TMRCA) for the new SIV sequences was inferred with the BEAST v1.6.2 program using a 308-bp alignment of all cdp of 89 SIV taxa, a relaxed molecular clock with an uncorrelated log normal rate distribution, a Yule tree prior, the HKY nucleotide substitution model with gamma distributed rates and an estimated proportion of invariable sites. TMRCAs were inferred by calibrating the molecular clock using an the estimated 10,000 year old separation of the drill (*Mandrill leucophaeus*) SIVs on mainland Africa from those on Bioko Island, Equatorial Guinea as previously described [36]. 50 million MCMC were used in each run and chain convergence and mixing, effective sample sizes (ESS), and Bayes Factors were determined using the program Tracer v1.5. All ESSs were greater than 400. Maximum clade credibility (MCC) trees were obtained using TreeAnnotator after a burn-in of the first 1000 trees. MCC trees were viewed using the program FigTree v1.3.1.

## Competing interests

The authors declare that they have no competing interests.

## Authors’ contributions

Conceived and designed the experiments: DHO, TLG, TCF, CAC. Performed the experiments: ML, SDS, WMS, AS, BT. Analyzed the data: ML, WMS. Wrote the paper: ML, WMS, TLG, DHO, TCF, NT. Conducted study in the field: DH, AT, GW, TLG. All authors read and approved the final manuscript.
